# Development of a Clinically-Precise Mouse Model of Rectal Cancer

**DOI:** 10.1371/journal.pone.0079453

**Published:** 2013-11-12

**Authors:** Hiroyuki Kishimoto, Masashi Momiyama, Ryoichi Aki, Hiroaki Kimura, Atsushi Suetsugu, Michael Bouvet, Toshiyoshi Fujiwara, Robert M. Hoffman

**Affiliations:** 1 AntiCancer, Inc., San Diego, California, United States of America; 2 Department of Surgery, University of California San Diego, San Diego, California, United States of America; 3 Division of Surgical Oncology, Department of Surgery, Okayama University Graduate School of Medicine, Dentistry and Pharmaceutical Sciences, Okayama, Japan; Stanford University, United States of America

## Abstract

Currently-used rodent tumor models, including transgenic tumor models, or subcutaneously growing tumors in mice, do not sufficiently represent clinical cancer. We report here development of methods to obtain a highly clinically-accurate rectal cancer model. This model was established by intrarectal transplantation of mouse rectal cancer cells, stably expressing green fluorescent protein (GFP), followed by disrupting the epithelial cell layer of the rectal mucosa by instilling an acetic acid solution. Early-stage tumor was detected in the rectal mucosa by 6 days after transplantation. The tumor then became invasive into the submucosal tissue. The tumor incidence was 100% and mean volume (±SD) was 1232.4 ± 994.7 mm^3^ at 4 weeks after transplantation detected by fluorescence imaging. Spontaneous lymph node metastasis and lung metastasis were also found approximately 4 weeks after transplantation in over 90% of mice. This rectal tumor model precisely mimics the natural history of rectal cancer and can be used to study early tumor development, metastasis, and discovery and evaluation of novel therapeutics for this treatment-resistant disease.

## Introduction

Heterotopic implantation models such as subcutaneous tumor implants in mice have been traditionally used to evaluate antitumor treatment because of their reproducibility and monitoring of tumor formation. However, heterotopic models do not have corresponding tumor microenvironments (TME) [1].

Genetically-engineered mouse models of cancer usually require a long time to develop tumors and are unpredictable with regard to frequency, time, and location of primary tumors and even more unpredictable when and where metastasis occurs. Since tumor development in these animals is rarely synchronous, a large cohort of animals must be housed in order to prepare for sufficient numbers of animals at appropriate stages of cancer. Large numbers of animals need to be examined over long periods of time to evaluate cancer treatment, and only survival is often used as an endpoint due to the difficulty of monitoring when tumors appear in internal organs [2].

Several kinds of models employing orthotopic implantation of tumors have been developed and utilized to mainly investigate antitumor drug efficacy [3]. In these models, either cancer cell suspensions are injected into the orthotopic organ or tumor tissue fragments are sutured on the corresponding organ [4], [5] with implantation of tissue fragments having been shown to be more accurate [5], [6]. With regard to colorectal cancer models, tumors have been established in submucosal tissues or on the colonic serosa, but not on the mucosal surface from which tumors actually arise in patients.

The aim of this study was to establish a “true” clinically-precise orthotopic rectal model in which tumors start forming in the mucosal tissue of the rectum.

## Materials and Methods

### Cell culture

The mouse colorectal cancer cell line CT26 and the human colorectal cancer cell line HCT-116 were cultured in RPMI 1640 medium supplemented with 10% FBS. CT26 is an N-nitroso-N-methylurethane-(NNMU)-induced mouse undifferentiated colon carcinoma cell line [7]. HCT-116 was derived from a primary human colon cancer specimen [8].

### GFP and RFP vector production

The pLEIN retroviral vector (Clontech), expressing enhanced green fluorescent protein (GFP) and the neomycin resistance gene on the same bicistronic message, was used as a GFP expression vector. PT67, an NIH3T3-derived packaging cell line, expressing the 10 Al viral envelope, was purchased from Clontech Laboratories, Inc. PT67 cells were cultured in DMEM supplemented with 10% FBS. For GFP vector production, PT67 packaging cells, at 70% confluence, were incubated with a precipitated mixture of DOTAP™ reagent (Boehringer Mannheim, Indianapolis, Indiana), and saturating amounts of pLEIN plasmid for 18 h. Fresh medium was replenished at this time. The cells were examined by fluorescence microscopy 48 h post-transduction. For selection, the cells were cultured in the presence of 500–2,000 µg/ml of G418 (Life Technologies, Grand Island, New York) for 7 d. The isolated packaging cell clone was termed PT67-GFP [9]–[12].

For RFP retrovirus production, the *Hind*III/*Not*I fragment from pDsRed2 (Clontech), containing the full-length RFP cDNA, was inserted into the *Hind*III/*Not*I site of pLNCX2 (Clontech) containing the neomycin-resistance gene. PT67 cells were cultured in DMEM supplemented with 10% FBS. For vector production, the PT67 packaging cells, at 70% confluence, were incubated with a precipitated mixture of LipofectAMINE reagent (Life Technologies) and saturating amounts of pLNCX2-DsRed2 plasmid for 18 h. Fresh medium was replenished at this time. The cells were examined by fluorescence microscopy 48 h post-transduction. For selection of a clone producing high amounts of RFP retroviral vector (PT67-DsRed2), the cells were cultured in the presence of 200 to 1,000 µg/mL G418 (Life Technologies) for 7 d. The isolated packaging cell clone was termed PT67-DsRed2 [9]–[12].

### GFP and RFP gene transduction of cancer cell lines

For GFP and RFP gene transduction, the cancer cells were incubated with a 1:1 precipitated mixture of retroviral supernatants of PT67-GFP or PT67-DsRed2 cells and RPMI 1640 containing 10% FBS for 72 h. Fresh medium was replenished at this time. Cells were harvested by trypsin/EDTA 72 h post-transduction and subcultured at a ratio of 1:15 into selective medium, which contained 200 µg/ml of G418. The level of G418 was increased up to 800 µg/ml in a stepwise manner. Clones expressing high levels of GFP or RFP were isolated with cloning cylinders (Bel-Art Products) using trypsin/EDTA and amplified by conventional culture methods in the absence of selective agent [9]–[12].

### Mice

Nude *nu/nu* mice (AntiCancer, Inc., San Diego, CA) were kept in a barrier facility at AntiCancer, Inc., under HEPA filtration and fed with autoclaved laboratory rodent diet (Tecklad LM-485, Western Research Products, Orange, CA). All animal studies were conducted with an AntiCancer Institutional Animal Care and Use Committee (IACUC)-protocol specifically approved for this study and in accordance with the principals and procedures outlined in the National Institute of Health Guide for the Care and Use of Animals under Assurance Number A3873-1. All animal procedures were performed under anesthesia using s.c. administration of a ketamine mixture (10 µl ketamine HCL, 7.6 µl xylazine, 2.4 µl acepromazine maleate, and 10 µl PBS).

### Nestin-driven green fluorescent protein (ND-GFP) nude mice

Interaction of the CT26-RFP tumor and host ND-GFP-expressing cells in the rectal mucosa was examined. CT26-RFP cells were orthotopically transplanted to ND-GFP nude mice [13] by the methods described below. Ten days after tumor transplantation, mice were sacrificed and the anorectum was dissected. Tumor and ND-GFP cell interaction was observed with the OV100 imaging system.

### Disruption of the rectal mucosal barrier

**Figure 1:**
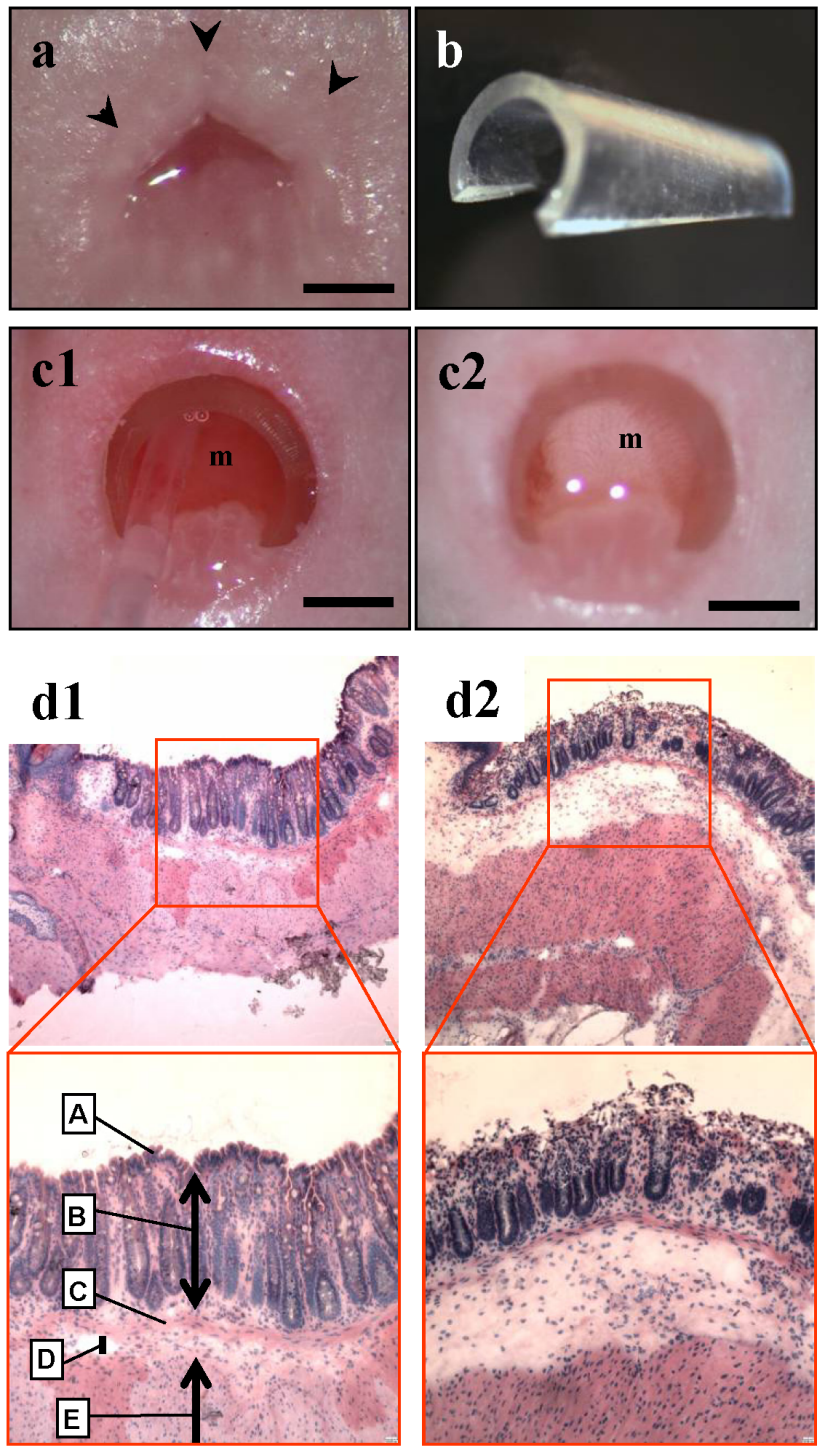
Disruption of the mucosal barrier of the rectum. (**a**) Normal appearance of mouse anus. Scale bar, 1 mm. (**b**) Retractor made from a drip infusion tube. (**c**) The anorectal lumen was dilated by inserting the retractor into the anorectum and instilled with an acetic acid solution. (**c1**) Before acetic acid preparation. (**c2**) The color of the rectal mucosa changed from reddish pink to whitish after treatment with acetic acid solution. m  =  rectal mucosa. Scale bar, 1 mm. (**d**) Histological examination just after acetic acid treatment showed that the epithelial cell layer of the rectal mucosa was traumatized. (**d1**) H&E section of normal anorectum. A  =  surface epithelium; B  =  mucosa; C =  muscularis mucosae; D  =  submucosa; E  =  muscularis externa. (**d2**) After acetic acid treatment. Note that only the upper part of the mucosa is disrupted. Top, × 40 magnification; bottom, × 100 magnification.

The rectal mucosal barrier was chemically disrupted by the following procedure: After mice were anesthetized, a polyethylene catheter was inserted into the rectal lumen through the anus. The rectal lumen was washed with 6 ml PBS to clean out the bowel contents. The rectal lumen was dilated by inserting a retractor into the anorectal region, and then the rectal mucosa was well soaked with 4% acetic acid solution for two minutes, followed by flushing with 6 ml PBS (Fig. 1).

### Intrarectal instillation of colorectal cancer cells and examination for tumor formation

Immediately after disruption of the rectal mucosal barrier, CT26-GFP cells (2.0×10^6^) in 50 µl Matrigel (BD Biosciences, San Jose, CA), were introduced and instilled on the rectal mucosal surface with a 28-gage needle inserted 4 mm from the anal ring. The anus was sealed, holding the retractor in the rectum, by plastic tape immediately after instillation of cancer cells to prevent cell leakage. The plastic tape sealing and the retractor in the rectum were removed from the mice after they recovered from anesthesia. After cancer-cell implantation, noninvasive observation with the OV100 Small Animal Imaging System [14] or the MVX-10 fluorescence microscope [15] (both from Olympus, Tokyo, Japan) was used to detect and characterize implanted cancer cells. Then mice were euthanized to explore possible metastasis and for histological studies. The tumor volume was calculated according to the following equation: Tumor volume (mm^3^)  =  length (mm) × width (mm) × depth (mm) × 0.5 [16].

### Fluorescence optical imaging and processing

The OV100 Imaging System and MVX-10 fluorescence microscope were used for fluorescence detection of the growing tumor. High-resolution images were directly captured on a PC and analyzed with CellR software (Olympus-Biosystems, Melville, NY) [14].

### Histological Examination

After mice were euthanized, the rectum was opened longitudinally and inspected for the presence of tumors. The excised rectal tumor was then embedded in freezing medium, frozen and sectioned at 7 µm with a cryostat. Slides were observed for tumor cell fluorescence and subsequently examined microscopically by hematoxylin-eosin staining.

## Results

### Disruption of the mucosal barrier of the rectum

The mucosal barrier of the rectum of nude mice was disrupted by treating the mucosa with 4% acetic acid solution (Fig. 1a-c). There were no deaths or overt toxicity related to this procedure. Histological examination revealed that the epithelial cell layer of the rectal mucosa was disrupted and peeled after acetic acid treatment (Fig. 1d). The mucosal tissue was now ready to accept the cancer cells.

### Orthotopic implantation of CT26-GFP cells

Immediately after chemical disruption of the rectal mucosal barrier, CT26-GFP mouse colorectal cancer cells, mixed in Matrigel, were introduced into the rectal lumen. The anus was sealed with plastic tape after filling the rectal lumen with the cancer-cell suspension, which ensured that the cancer cells remained on the rectal mucosa during anesthesia. The total procedure time was approximately 15 min per mouse, and there were no complications or deaths related to both acetic acid treatment and cancer-cell implantation.

**Figure 2:**
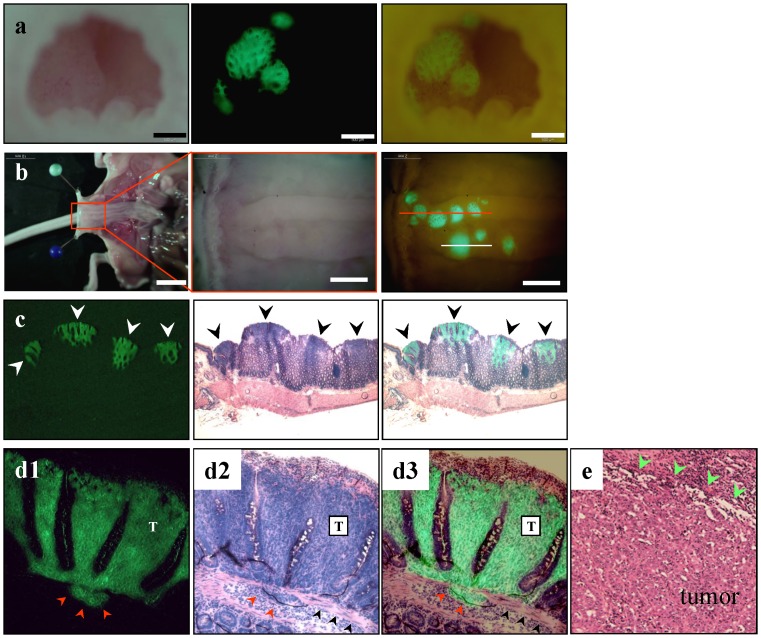
Intramucosal CT26-GFP tumor formation in the mouse rectum. (**a**) The rectum was imaged noninvasively 10 days after implantation. Left, brightfield observation; middle, CT26-GFP tumors growing on the rectal mucosa were clearly visible under fluorescence observation; right, simultaneous observation under bright-light and fluorescence imaging. Scale bar, 500 µm. (**b**) After laparotomy, the rectum was opened longitudinally from the anterior wall. Left, gross appearance of the abdominal cavity. Scale bar: 10 mm; middle, detail of the boxed region; right, simultaneous observation under bright-light and fluorescence. The location of tumor formation was limited on the posterior wall of the terminal rectum. Red line, the direction of rectal cross-section of (**c**). White line, the direction of tumor cross-section of (d). Scale bar, 2 mm. (**c**) Histological analysis confirmed that GFP-positive lesions were medullary-type adenocarcinomas with no glandular structures. Left, fluorescence detection of tumors in a frozen section; middle, GFP-positive tumors showed high cellularity and were confirmed as tumors growing in the mucosal layer of the rectum; right, merged image of H&E histological section and fluorescence detection. Note that cancer cells locate only in the mucosal layer of the rectum. (**d1-3**) CT26 medullary-type adenocarcinomas invading the submucosal layer beyond the limits of the muscularis mucosae (red arrow heads). T  =  tumor. Black arrow heads, muscularis mucosae. (**e**) Histological appearance of human medullary-type adenocarcinoma. Green arrow heads indicate tumor edge [23].

After cancer-cell implantation, the rectal lumen was noninvasively examined with the MVX-10 fluorescence microscope or OV100 Small Animal Imaging System (Fig. 2a). The CT26-GFP cells on the rectal mucosa could be readily imaged by GFP fluorescence beginning 6 days after implantation at the latest. The incidence of rectal tumors in the rectal mucosa was 100%. Neither instillation of cancer cells alone or acetic acid treatment alone resulted in growth of a rectal tumor.

As shown in Fig. 2b, the location of tumor formation was limited to the posterior wall of the terminal rectum. This limitation of tumor formation might be due to allowing the cancer-cell suspension to contact only the disrupted dorsal mucosa of the rectum.

At 10 days after implantation, fluorescence microscopy of frozen sections and histological analyses revealed that tumors were growing in the mucosal layer in a similar fashion to human medullary colon carcinoma (Fig. 2c-e). Around this time, CT26-GFP cells invading the submucosal layer, beyond the limits of the muscularis mucosae, were also detected (Fig. 2d).

**Figure 3:**
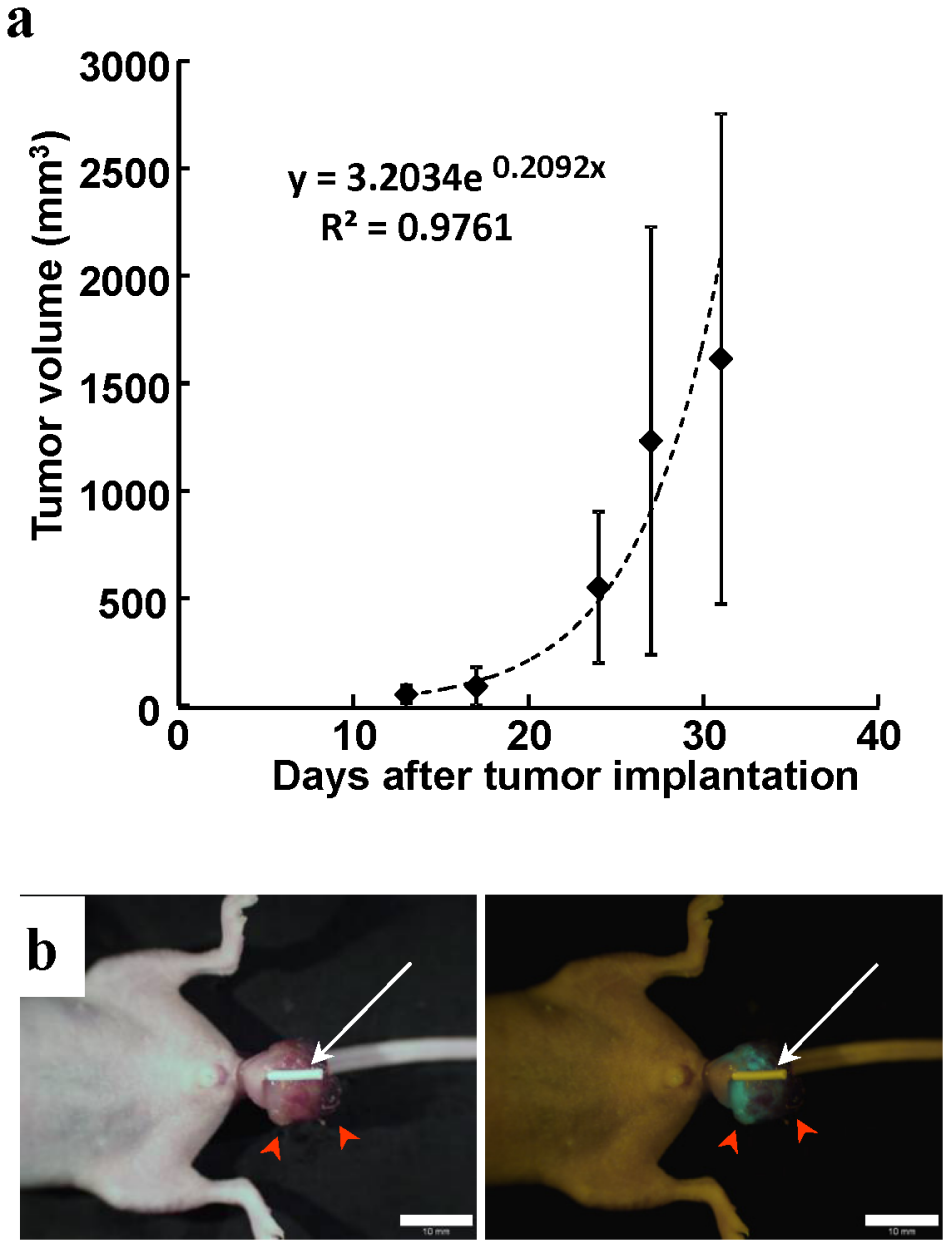
Growth kinetics of CT26-GFP rectal tumors in mice. (**a**) All the mice eventually had only a single tumor formed on the rectal mucosa. Tumor size increased over time. (**b**) Prolapsed CT26 rectal tumor growing outside of the anus at 4 weeks after implantation (red arrow heads). White tube inserted into the rectum (white arrow) shows that anorectal passage is well preserved, and there is no obstruction. Scale bar, 10 mm

All the mice eventually had only a single tumor formed on the rectal mucosa, and the tumor size gradually increased over time (Fig. 3a). Since the rectal tumors prolapsed through the anus when they reached approximately 3 mm in size and then grew outside of the anus, no rectal obstruction occurred during the whole observation period in any of the mice (Fig. 3b).

**Figure 4:**
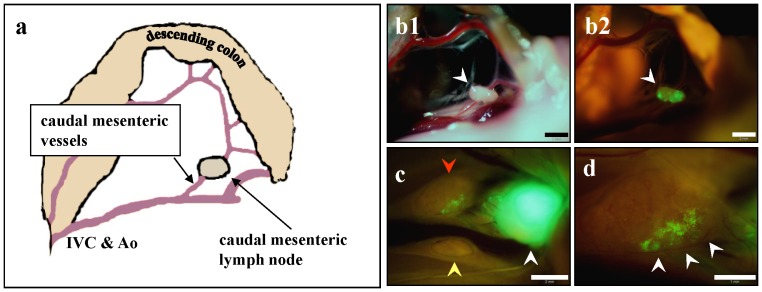
Spontaneous lymph nodes metastasis. (**a**) Schematic drawing of the anatomy in (**b**). The caudal mesenteric lymph node at the origin of the caudal mesenteric artery. This lymph node is equivalent to the inferior mesenteric lymph node in humans. Scale bar, 2 mm. (**b1**) Caudal mesenteric lymph node under bright-light observation. (**b2**) GFP fluorescence of the caudal mesenteric lymph node in (**b1**), indicating that the lymph node contained a metastasis. Scale bar, 2 mm. (**c**) Two para-aortic lymph nodes (red and yellow arrows) were identified in another mouse. White arrow indicates metastasized caudal mesenteric lymph node. Scale bar, 2 mm. (**d**) Higher magnification observation of a para-aortic lymph node indicated by red arrow in (**c**). GFP fluorescence of a micro-metastasis was detected (white arrows). Scale bar, 1 mm.

**Figure 5:**
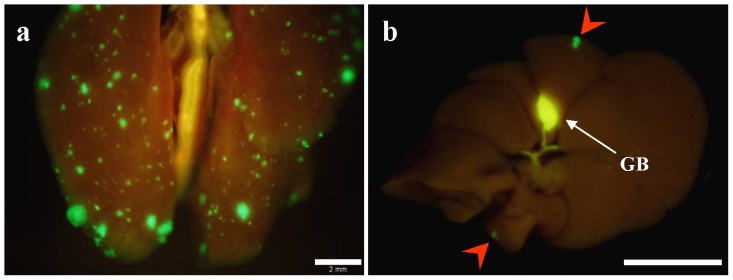
Spontaneous hematogenous metastasis. (**a**) Macroscopic appearance of lung. Lung metastatic foci were detected with GFP fluorescence. Scale bar, 2 mm. (**b**) Macroscopic appearance of the liver. Fluorescence imaging detected GFP expression of CT26-GFP liver metastases (red arrows). Scale bar, 10 mm.

At 4 weeks after instillation of cancer cells, the mean tumor volume (mean volume ± SD) reached 1232.4±994.7 mm^3^ (Fig. 3a), and spontaneous lymph node metastases were detected with high occurrence (Fig. 4). The metastatic rate to the caudal mesenteric lymph node (inferior mesenteric lymph node in humans) [17], [18], which is a regional lymph node in rectal cancer, was 90.9%. Metastasis to the para-aortic lymph nodes was 54.5% (Table 1). Spontaneous lung metastasis was also detected in 91.8% of mice (Fig. 5). However, only 9% of the mice (1/11) had liver metastasis (Table 2).

**Table 1:** Lymph node metastasis of the rectal tumor.

Lymph node (LN)	Number of metastatic LN/mouse (range)	Metastatic rate (%)
Caudal mesenteric LN	0–1	90.9
Para-Aortic LN	0–2	54.5

**Table 2:** Hematogenous metastasis of the rectal tumor.

Organ	Number of metastatic tumor foci/mouse (range)	Metastatic rate (%)
Liver	0–2	9.0
Lung	0–219	91.8

At approximately 4 weeks, mice showed cachectic symptoms and had to be sacrificed because of tumor size.

### Angiogenesis of mouse rectal tumor orthotopically implanted in ND-GFP nude mice

**Figure 6:**
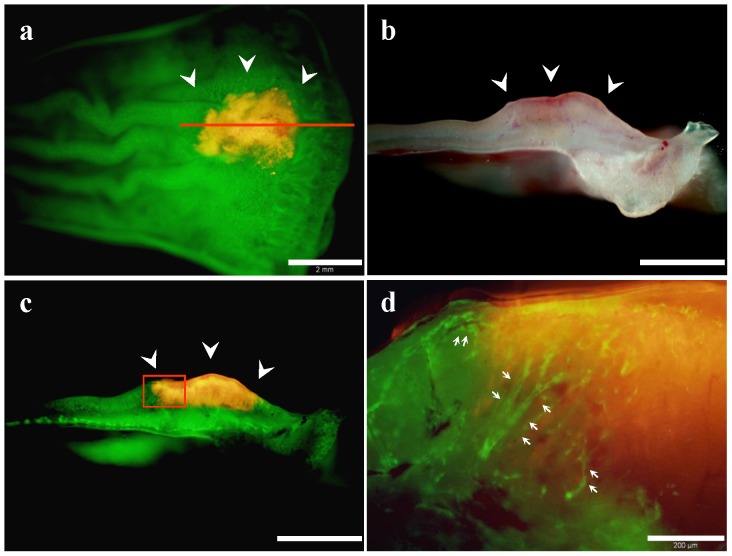
Dual-color imaging of tumor–host interaction in nestin-driven green fluorescent protein (ND-GFP) nude mice. (**a**) CT26-RFP tumor growing in the rectal mucosa of ND-GFP nude mice (white arrow heads). Red line, the direction of the rectal tumor cross-section of (**b**). Scale bar, 2 mm. (**b**) Cross-section of the rectal tumor. Bright- light observation. Scale bar, 2 mm. (**c**) Fluorescence observation of (**b**). Scale bar, 2 mm. (**d**) Detail of the boxed region in (**c**). Host-derived ND-GFP-expressing blood vessels were visualized in the RFP-expressing CT26 rectal tumor in the ND-GFP nude mouse (white arrows). Scale bar, 200 µm.

RFP-expressing CT26 cells were orthotopically implanted into the rectum of nestin-driven GFP (ND-GFP) nude mice by the methods described above. Ten days after implantation, ND-GFP–expressing nascent blood vessels were visualized growing into the RFP-expressing CT26 rectal tumor in the ND-GFP nude mouse (Fig. 6).

### Tumor formation by rectal instillation of human of cancer cells in nude mice

The applicability of this implantation method was evaluated with human cancer cells. With the same technique, human colon cancer HCT-116-RFP cells also formed rectal tumors in nude mice.

## Discussion

Clinically-accurate animal models in which tumors arise from early-stage and later become invasive and metastatic would be of great value in order to predict clinical outcomes for cancer treatment more precisely. Few transgenic models of invasive colorectal cancer exist [4]. The *Apc* ^Min+/−^ mouse model typically develop adenomas but not invasive adenocarcinomas [19]. Additionally, tumor development in genetically-engineered mice is rarely synchronous, which makes it difficult to prepare sufficient numbers of animals at appropriate stages for cancer treatment evaluation [2].

It has been demonstrated that orthotropic implantation allows the growth and metastatic potential of the transplanted tumors to be expressed and reflect clinical cancer [5], [20]. Many colorectal cancer models employing orthotropic implantation have been also reported. However, in these models, either a cancer cell suspension was injected into the submucosal layer of the colorectum [4], [5], [21], or tumor tissue fragments were simply sutured on the colon serosa. In these models, tumors grow in the submucosal tissues leaving the mucosa (which is the true origin of colorectal cancer) intact or from the beginning tumors grow only on the serosal surface, which is equivalent to advanced stage colorectal cancer in the clinic.

The aim of the present study was to establish a clinically-accurate orthotopic colorectal model in which tumors start forming in the mucosal tissue. We hypothesized that precise orthotopic implantation could be achieved with chemical disruption of the mucosal surface barrier of the rectum and subsequent installation of CT26 cancer cells.

Fluorescently-labeled cancer cells [22] enabled highly sensitive, real-time, and noninvasive detection of very early-stage cancer on the rectal mucosa and micro-metastatic foci in the lymph nodes and the lung, which would be otherwise undetectable in live tissue or even histological examination [20].

The location of primary tumor formation was limited to the posterior wall of the terminal rectum, which is the most convenient observation point in the mouse colorectum. The reproducibility of tumor location allowed easier and more focused noninvasive examination over time. The histological appearance of the tumor was very similar to human colon cancer classified as undifferentiated or medullary-type adenocarcinoma [23].

Lymph node metastases and lung metastasis were detected in the mice implanted with the CT26-GFP cells with high incidence, which could be considered as “true” spontaneous metastasis, since in this method it is highly unlikely that cancer cells can be directly introduced to the venous or lymphatic circulation artificially at the time of implantation [4]. Though liver is a most common metastatic site of colorectal cancer, liver metastasis was found in only 9% of mice (1/11) in this model. This may be explained by the fact that in this model, tumors form at the lower part of the rectum, blood from which drains into the systemic venous circulation dominantly over the portal venous circulation [24]. Rectal cancer is more likely than colon cancer to result in lung metastases without liver metastases in the clinic [24].

Unlike the cecal injection model, this model does not require laparotomy, through which unwanted peritoneal dissemination is caused by cancer cells dissociated at the time of the transplantation [4]. The current method is also applicable in immunocompetent hosts with cancer cell lines of mouse origin and would be suitable for tumor immunology studies.

In ND-GFP nude mice, GFP expression is under the control of the of the nestin promoter [13], [25] in which host-derived ND-GFP-expressing blood vessels were visualized in the early stage rectal tumors formed from CT26-RFP cells.

To our knowledge, this is the first model that can reliably provide truly orthotopic rectal cancer growing in the mucosal tissues, and later cause spontaneous lymph node and lung metastasis which are readily detectable by GFP fluorescence with high sensitivity. This model could be used for studying early events in tumor growth or assessing intraluminal factors (dietary compounds, bile acid, intestinal microflora, etc.), that may function to enhance or inhibit the growth of colorectal cancer in early stage cancer.

In conclusion, implantation of cancer cells to the mucosal tissue enabled by the techniques described in the present report allowed the tumors to grow in a manner which mimics clinical human disease. This model would be useful for evaluation of the therapeutic efficacy of antitumor drugs, and may be able to be utilized to study early events in the TME.
